# Exploring equity and inclusion in Malawi’s National Disability Mainstreaming Strategy and Implementation Plan

**DOI:** 10.1186/s12939-020-01378-y

**Published:** 2021-01-07

**Authors:** Ikenna D. Ebuenyi, Emma M. Smith, Alister Munthali, Steven W. Msowoya, Juba Kafumba, Monica Z. Jamali, Malcolm MacLachlan

**Affiliations:** 1 Assisting Living & Learning (ALL) Institute, Department of Psychology, Maynooth University, Maynooth, Ireland; 2 Centre for Social Research, University of Malawi, Zomba, Malawi; 3 Independent Consultant in Disability and Development, Blantyre, Malawi; 4 Olomouc University Social Health Institute (OUSHI), Palacký University, Olomouc, Czech Republic

**Keywords:** Equity, Inclusion, Persons with disabilities, EquIPP, Malawi, Policy process

## Abstract

**Background:**

Equity and inclusion are important principles in policy development and implementation. The aim of this study is to explore the extent to which equity and inclusion were considered in the development of Malawi’s National Disability Mainstreaming Strategy and Implementation Plan.

**Methods:**

We applied an analytical methodology to review the Malawi’s National Disability Mainstreaming Strategy and Implementation Plan using the EquIPP (Equity and Inclusion in Policy Processes) tool. The EquIPP tool assesses 17 Key Actions to explore the extent of equity and inclusion.

**Results:**

The development of the Malawi National Disability Mainstreaming Strategy and Implementation Plan was informed by a desire to promote the rights, opportunities and wellbeing of persons with disability in Malawi. The majority (58%) of the Key Actions received a rating of three, indicating evidence of clear, but incomplete or only partial engagement of persons with disabilities in the policy process. Three (18%) of the Key Actions received a rating of four indicating that all reasonable steps to engage in the policy development process were observed. Four (23%) of the Key Actions received a score five indicating a reference to Key Action in the core documents in the policy development process.

**Conclusions:**

The development of disability policies and associated implementation strategies requires equitable and inclusive processes that consider input from all stakeholders especially those whose wellbeing depend on such policies. It is pivotal for government and organisations in the process of policy or strategy development and implementation, to involve stakeholders in a virtuous process of co-production – co-implementation – co-evaluation, which may strengthen both the sense of inclusion and the effectiveness of the policy life-cycle.

## Background

Equity and inclusion are important principles in policy development and implementation [1, 2]. Equity refers to ensuring the absence of systemic and structural disparities between individuals or groups whereas inclusion entails the extent to which individuals or groups feel a part of an organization or process [2–4]. The UN Convention on the Rights of Persons with Disabilities (CRPD) highlights the importance of equity and inclusion for participation of persons with disabilities [5]. Inclusive laws and policies are very important in safeguarding equity and inclusion of persons with disabilities and vulnerable populations in society [3].

In most societies, persons with disabilities and other vulnerable populations face overt and covert barriers which hinder their participation in processes affecting their lives [6]. These barriers are sometimes systemic, structural, and context dependent. Despite the recommendations of the CRPD for State Parties to safeguard the rights of persons with disabilities to public services such as education, health, and employment, the protection and guarantee of rights of persons with disabilities is often still perceived as charity and goodwill [7]. Even in settings with a high index of social inclusion, it is not uncommon for persons with disabilities to be denied participation in the decision-making processes that affect their lives [6]. These experiences of social exclusion mean important stakeholders with relevant experiential knowledge do not contribute to development processes or policy formulation. Consequently, policies may not address the realities faced by these individuals on a day-to-day basis and may even serve to further marginalize this already marginalized group.

The Organisation for Economic Co-operation and Development (OECD) suggests that in order to achieve equitable and inclusive outcomes, policies ought to adopt equitable and inclusive processes [1]. This is very important in policy development and, implementation and evaluation. Studies suggest laws and policies often abound; however, what is often lacking is their actual implementation [7]. However, policy implementation is not independent of the formulation processes and mechanisms set in place in an inclusive manner to ensure its acceptability. Huss and MacLachlan argue the inclusion process must entail procedural and substantive inclusion wherein there is a deliberate action of government to include the interests of vulnerable and marginalized groups in policy processes [2]. Hence, inclusion is more than mere participation but involves continuous engagement and *coproduction* with stakeholders in the policy process [2].

In Malawi, the government is committed to promotion of an inclusive society and this prompted their development of the National Disability Mainstreaming Strategy and Implementation Plan (NDMS&IP) in 2018 to bridge the gap between policy and practice [8]. The NDMS&IP was perceived as integral to eliminating discrimination against persons with disabilities and increasing their access to basic social services [8]. However, despite the NDMS&IP and other national policies on disability, access to services and assistive products continues to elude persons with disabilities in Malawi [9–11]. Considering the good intention for developing the NDMS&IP, it is not clear how inclusive the process of its development was and the extent persons with disabilities contributed.

The aim of this study was to explore equity and inclusion in Malawi’s National Disability Mainstreaming Strategy and Implementation Plan using the EquIPP (Equity and Inclusion in Policy Processes) tool. This study is part of the larger Assistive Product List Implementation Creating Enablement of inclusive SDGs (APPLICABLE) project that seeks to develop a framework for creating an effective national Assistive Technology (AT) policy and specify a system capable of implementing that policy, in Malawi [12]. The results from this study will guide and provide information on how to integrate equity and inclusion in the proposed development of an AT policy and or strategy for Malawi.

## Methods

We adopted a retrospective analytical review methodology to review the Malawi National Disability Mainstreaming Strategy and Implementation Plan [8] using the EquIPP tool [2]. The EquiPP tool measures the extent to which equity and inclusion were adopted in the process of policy development, implementation and evaluation [2]. This tool has been previously used in the review of Malawi’s National HIV and AIDS Policy [13].

### The Malawi National Disability Mainstreaming Strategy and Implementation Plan (NDMS&IP)

Although Malawi has a National Policy on Equalisation of Opportunities for Persons with Disabilities (NPEOPWD) developed in 2006 [14], the NDMS&IP was developed to bridge the gaps between policy and practice in disability. Hence, the NDMS&IP was an attempt by the government to develop a strategy for promoting inclusion of disability issues in sectoral policies and strategies.

The NDMS&IP was a product of the joint effort of various stakeholders including the Department of Disability and Elderly Affairs (DDEA) in the Ministry of Gender, Children, Disability and Social Welfare (MGCDSW). In addition, there were stakeholders and technical experts from government ministries (Health, Education, Science and Technology, Labour and Manpower Development), local and international NGOs and persons with disabilities drawn from the Federation of Disability Organisations in Malawi (FEDOMA). The process was consultative and included a stakeholder consultation workshop.

### The EquIPP framework

The EquIPP framework was developed to support Equity & Inclusion equity and inclusion in the processes of policy development, implementation and evaluation in diverse policy contexts including low, middle- and high-income settings. It was developed between November 2014 – February 2016 through a literature review of stakeholder methodologies to equity and social inclusion. The process was iterative and involved multiple rounds of stakeholder consultations and revisions using feedback from both high- and low-income settings.

It consists of 17 Key Actions expected to guide and ensure equitable and inclusive policy processes. The 17 Key Actions are sub-divided into nine themes: inclusive and participatory policy procedure; cross-sectoral and intergovernmental cooperation and coordination; matching social need and provision; social budgeting, inclusive and responsive implementation; implementation partnerships and cooperation; multi-dimensional and context driven data collection; data-fit-for-purpose and comprehensive and inclusive dissemination system [2]. The tool was developed in collaboration with the UNESCO Management of Social Transformation (MOST) (Management of Social Transformation) programme and the United Nations Partnership for the Rights of Persons with Disabilities (UNPRPD) programme. The process entailed extensive literature review, review of other instruments and expert opinion from across UN agencies and civil society [2]. The tool uses an assessment matrix (checklist) to assess the extent to which policy processes qualify as equitable and inclusive. It applies the Policy Engagement Key Action Scale (PEKAS) [2] which is a 7-point scale (0–7) (Table 1) to assess the 17 Key Actions. The assessment can be done in real time or retrospectively.

**Table 1:** Policy Engagement Key Action Scale (PEKAS) assessing the extent to which engagement with stakeholders has been a central element of the policy development and/or implementation process

Pol Policy Engagement Key Action Scale (PEKAS)	Rating
**Absent** – no evidence it has been considered	0
**Recognition** – evidence of awareness but no associated action	1
**Minor action** – evidence of token or minimal efforts to engage	2
**Moderate action** – evidence of clear but incomplete or partial engagement	3
**Comprehensive action** – evidence that all reasonable steps to engage have been taken	4
**Policy evaluation**– reference to Key Action in core document(s)	5
**Process Evaluation** – evidence gathered from diverse stakeholders of satisfaction with the process of engagement	6
**Outcome Evaluation** - evidence gathered from diverse stakeholders of satisfaction with the outcomes of engagement	7

### Analysis

We conducted the analytical review process in three stages. In the first stage, two independent researchers (IDE and EMS) reviewed the NDMS&IP using the EquIPP tool across the 17 Key Actions. Thereafter, the raters met to discuss and resolve the differences in rating.

In the second stage, the results of the review and rating were shared with two of the co-authors (A M and SWM) who are experts in disability research and policy in Malawi and had first-hand knowledge of the development and implementation of the NDMS&IP. We drew on their knowledge for the analysis, particularly where there were questions which could not be resolved through evaluation by the independent reviewers.

In the third stage, the results of the analysis from the researchers were presented to the Action Research Group (ARG) the APPLICABLE project [12]. The ARG consist of 15 stakeholders purposively selected from users and providers of AT and experts on disability in Malawi. The ARG are leading on the process of the policy development in collaboration with the research team [12]. The feedback and recommendations from the ARG were incorporated into the results presented herein.

## Results

**Fig. 1:**
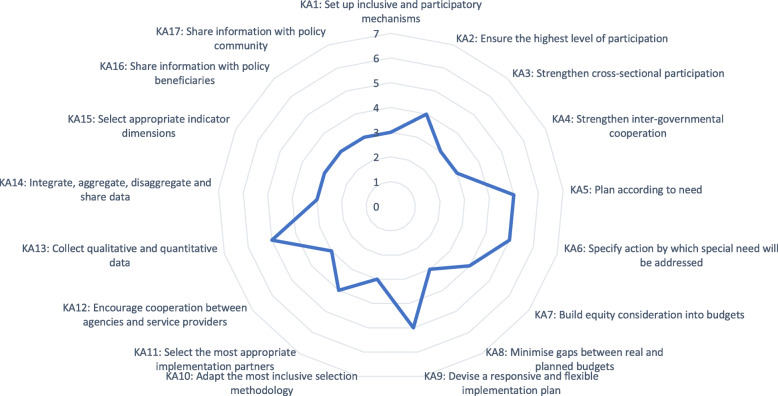
Summary of EquIPP Scores for the Malawi National Disability Mainstreaming Strategy and Implementation Plan

Figure 1 illustrates the summary of EquIPP scores for the Malawi NDMS&IP according to the 17 Key Actions. The majority (58%) of the Key Actions received a rating of three indicating evidence of clear but incomplete or partial engagement of persons with disabilities in the policy process. Three (18%) of the Key Actions received a rating of four indicating that all reasonable steps to engage in the policy development process were observed. Four (23%) of the Key Actions received a score five indicating a reference to Key Action in the core documents in the policy development process. Table 2 highlights the Key Actions, the Themes and the corresponding rating. In Key Action two, it was unclear if stakeholders participated in the outcome evaluation. In Key Action five, we found that while the role of the local government or councils was defined, their mode of action was absent. Similarly, in Key Action 15, indicators for priority areas were mentioned but it is not known whether indicators were chosen with all legitimate stakeholders participating. Although, Key Actions 16 and 17 indicated that reference was made to dissemination, it was unclear how this would be conducted with stakeholders.

**Table 2:** Key Actions, Themes and Rating of the Malawi National Disability Mainstreaming Strategy and Implementation Plan using EquIPP

Key Action	Theme	Rating
KA1: Set up inclusive and participatory mechanisms	T1: Inclusive and participatory policy procedure	3
KA2: Ensure the highest level of participation		4
KA3: Strengthen cross-sectional participation	T2: Cross-sectoral and intergovernmental cooperation and coordination	3
KA4: Strengthen inter-governmental cooperation		3
KA5: Plan according to need	T3: Matching social need and provision	5
KA6: Specify action by which special need will be addressed		5
KA7: Build equity consideration into budgets	T4: Social budgeting	4
KA8: Minimise gaps between real and planned budgets		3
KA9: Devise a responsive and flexible implementation plan	T5: Inclusive and responsive implementation	5
KA10: Adapt the most inclusive selection methodology		3
KA11: Select the most appropriate implementation partners	T6: Implementation partnerships and cooperation	4
KA12: Encourage cooperation between agencies and service providers		3
KA13: Collect qualitative and quantitative data	T7: Multi-dimensional and context driven data collection	5
KA14: Integrate, aggregate, disaggregate and share data	T8: Data-fit-for -purpose	3
KA15: Select appropriate indicator dimensions		3
KA16: Share information with policy beneficiaries	T9: Comprehensive and inclusive dissemination system	3
KA17: Share information with policy community		3

The NDMS&IP stated that key stakeholders, such as persons with disabilities and their umbrella organisations, were involved in its development. It was also stated that the process was inclusive and consultative to the extents rated above. Also, FEDOMA played a key role on behalf of persons with disabilities during the development of the NDMS&IP [8]. However, feedback from the ARG suggested that sometimes the umbrella organisations do not represent users or persons with disabilities. Also, they proposed that policy and strategy development for persons with disabilities must engage the direct users of services in the communities.

## Discussion

Our review indicates that the development of the Malawi NDMS&IP was motivated by a desire by the government to evolve an inclusive policy document for the welfare and wellbeing of persons with disabilities [8]. We fully acknowledge that there is no perfect policy process and that sometimes competing agendas and the exigencies of time mitigate against policy processes being as inclusive as those who oversee them would wish them to be. In this context we identify some elements where the policy development process or implementation could be strengthened. It is heartening to note that “social inclusion” is regularly identified as a focus within the document and is one of the six priority areas of the NDMS&IP. While it is reassuring that none of the Key Actions received a rating of zero, implying no evidence of it being considered, it is also concerning that none of the Key Actions received a score of six or seven that would reveal evidence that diverse stakeholders were satisfied with the process and outcome of the engagement. Without this sort of follow-up engagement there is a danger that policy development is seen as a statutory obligation rather than a commitment to implement in partnership with key stakeholders. Considering that service provision to persons with disabilities in Malawi relies on non-state actors as much as on government institutions the ethos of *coproduction* must be matched with a *co-implementation* ethos, and a commitment to assessing the consequences of doing this is essential. As this is a relatively new document there is still considerable scope for a fully engaged co-implementation and its *co-evaluation*.

Over half of the Key Actions received a score of three that showed minimal or partial engagement of stakeholders. In particular, themes *two, eight* and *nine* had homogenous scores of three across all relevant Key Actions. This suggests that achieving cross-sectoral and intergovernmental cooperation and coordination, and having data fit for purpose, with effective dissemination may not be easily achievable; perhaps on account of the partial engagement. Despite the listing of many government ministries and their diverse roles in the implementation of the NDMS&IP, it is not apparent how these important stakeholders would help fulfil anticipated objectives. Studies indicate that effective stakeholder engagement helps in achieving desired goals and objectives [4, 15]. This understanding justifies the recommendations for transdisciplinary research approaches in identifying pathways to change regarding persistent societal problems [15]. In order to ensure equity, inclusion and effectiveness in programmes for persons with disabilities, we cannot continue doing things *for* persons with disabilities, *but with them*. This is especially relevant in relation to the observation by the ARG that sometimes umbrella organisations may not represent the ‘actual interest or position’ of persons with disabilities in communities or rural settings. Hence, equitable policy development must consider persons with disabilities in the communities and engage them in both the development of policy and implementation strategies.

It is commendable that four of the Key Actions received scores of five showing a reference to important documents in the policy development process. This is particularly interesting in themes *three* and *seven* related to matching social need and provision, and multi-dimensional and context driven data collection, respectively. Matching social needs and provision aligns to the recommendations of the United Nations Development Programme for policy objectives to correspond to the specific needs of vulnerable populations [16]. This may help in achieving equity and fulfil the recommendations of the Sustainable Development Goals to leave no one behind [17]. Similarly, the use of context driven data that combines quantitative and qualitative data promotes the use of participatory evidence in the policy development process [16]. Participatory evidence ensures data is decentralized and empowers stakeholders by including context rich information [16].

### Limitations

The extent to which persons with disabilities in Malawi were involved in the development of the NDMS&IP may best be answered by the individuals in these group who were present in Malawi at that time. What we have attempted to do in this review is to highlight the need to ensure equity and inclusion in the process of development and implementation of policies or strategic plans, with all stakeholders in Malawi and indeed globally.

This study was a retrospective desk review, and therefore did not include persons with disabilities living in Malawi, nor government officials involved in the process. However, as argued by Huss and MacLachlan [2], it is our contention that processes to engage with and include marginalized groups must be explicit, documented and open to public scrutiny. Our analysis has therefore been based on the analysis of documentation available and a few stakeholders in the disability sector. While we are aware that it may not have been possible to document all the activities undertaken during the development of the NDMS&IP on which we relied to conduct the review, we believe it is important for third party researchers to be able to conduct independent analysis of policy developments and implementation processes.

## Conclusions

The development of the Malawi NDMS&IP was informed by a desire by the government to promote effective disability mainstreaming for an inclusive society. The extent to which the process involved persons with disabilities is not clear. Disability and policy development require equitable and inclusive processes that considers input from all stakeholders. Despite the good intentions of the policy developers, it may not achieve desired objectives without input from stakeholders with lived experiences. The involvement must go beyond the leadership of disability specific organisations and of umbrella organisations of persons with disabilities to ensure that the voices of ordinary persons with disabilities including those in rural areas are also heard. It is pivotal for governments, policy makers and organisations in the process of policy development to involve such stakeholders from the onset in all the processes related to the policy or strategy, development and implementation. There remains the opportunity to engage with organisations of people with disability (OPDs) for the implementation and evaluation of the Malawi National Disability Mainstreaming Strategy and Implementation Plan. Without such engagement effective implementation will be undermined.

## Data Availability

The datasets used in the current study are available from the corresponding author on reasonable request.
